# [Retracted] Rosiglitazone exerts neuroprotective effects via the suppression of neuronal autophagy and apoptosis in the cortex following traumatic brain injury

**DOI:** 10.3892/mmr.2026.13894

**Published:** 2026-04-29

**Authors:** Junchao Yao, Kebin Zheng, Xiang Zhang

Mol Med Rep 12: 6591–6597, 2015; DOI: 10.3892/mmr.2015.4292

Following the publication of the above paper, it was drawn to the Editor's attention by a concerned reader that various of the western blot data featured in Fig. 2B appeared to have also been included in Figs. 4B and 8A; moreover, western blot data also appeared to have been duplicated comparing Figs. 3A and 8A, Figs. 4B and 5A, Figs. 3A and 5A, and Figs. 6A and 7A. In addition, western blot data featured in various of these figures also reappeared in two papers written by different authors at different research institutes that were submitted for publication at later dates to the journals *Experimental and Therapeutic Medicine* and *Molecular Medicine Reports.*

In view of the fact that so many instances of the duplication of data within several of the figures in the above paper were identified, the Editor has decided that this paper should be retracted from the Journal on account of a lack of confidence in the data. The authors were asked for an explanation to account for these concerns, but the Editorial Office did not receive a reply. The Editor apologizes to the readership for any inconvenience caused.

